# 应用多肽芯片研究非小细胞肺癌患者血清中的EGFR自身抗体

**DOI:** 10.3779/j.issn.1009-3419.2010.07.13

**Published:** 2010-07-20

**Authors:** 元 李, 文涛 岳, 玥 王, 丽娜 张, 勐 顾, 绍发 许

**Affiliations:** 1 101149 北京，北京市结核病胸部肿瘤研究所，细胞生物学研究室 Department of Cellular and Molecular Biology, Beijing TB and Thoracic Tumor Research Institute, Beijing 101149, China; 2 101149 北京，北京市结核病胸部肿瘤研究所，北京胸科医院胸外科 Department of Thoracic Surgery, Beijing Chest Hospital, Beijing 101149, China

**Keywords:** 肺肿瘤, EGFR, 多肽芯片, 自身抗体, Lung neoplasms, EGFR, Peptide array, Autoantibody

## Abstract

**背景与目的:**

自身抗体作为新的肿瘤标志物在肺癌的早期诊断和预后评价中可能发挥重要作用, 本研究利用多肽芯片检测非小细胞肺癌患者血清中表皮生长因子受体（epidermal growth factor receptor, EGFR）的自身抗体, 并筛选自身抗体识别的抗原表位。

**方法:**

使用Intavis公司ASPSL多肽芯片合成仪合成EGFR多肽芯片, 利用多肽芯片检测非小细胞肺癌患者血清中EGFR自身抗体, 并筛选自身抗体识别的抗原表位。结果使用EGFR多肽芯片检测了20例非小细胞肺癌患者,

**结果:**

有6例阳性, 阳性率为30%, 在该6例阳性患者中发现了9个高频位点, 并且有8个高频位点集中在EGFR胞外段的第Ⅲ和第Ⅳ结构域。

**结论:**

本研究为我们进一步研究EGFR和EGFR自身抗体的功能提供了新的线索。

## Abstract (en)

**Background and objective:**

Autoantibodies as new tumor markers may play an important role in the early diagnosis and evaluating the prognosis of lung cancer. In this study, we detect epidermal growth factor receptor (EGFR) autoantibodies using peptide array and screen the epitopes which are recognized by EGFR autoantibodies.

**Methods:**

Peptide array covering the extracellular domain of EGFR protein was synthesized by a synthesizer (ASPSL) made by Intavis company. EGFR autoantibodies in the serums of non-small cell lung cancer patients was detected using peptide array.

**Results:**

Six of 20 patients were found to have EGFR autoantibodis. The positive rate is 30%. Nine high frequency spots were found in the 6 positive patients and 8 high frequency spots clustered in the Ⅲ and Ⅳ domains.

**Conclusion:**

These findings will offer new clues for the futher studies of EGFR and EGFR autoantibodies.

肺癌是全球范围内最常见和最致命的恶性肿瘤之一，对人类的生命和健康构成严重威胁。研究^[1]^显示临床上无法发现的早期肿瘤即可以出现血清中自身抗体阳性，因而血清自身抗体有望作为肺癌早期诊断的分子标志。目前常用ELISA方法进行血清自身抗体检测，但是传统的ELISA方法使用蛋白作为抗原，只能检测到血清中有无自生抗体而并不能确定抗体所识别的抗原表位，因而临床应用意义有限，而目前抗原表为已被广泛地应用于疾病诊断﹑预后评价和疫苗的研发^[2-4]^。多肽芯片是近年发展起来的一种新技术，所谓多肽芯片就是将蛋白质分解成肽段合成到纤维素膜上组成密集的点阵，与传统的ELISA方法相比，多肽芯片不仅能检测到血清中的自身抗体，而且能够确定自身抗体所识别的抗原表位，为进一步研究自身抗体的功能提供线索，因此多肽芯片已被广泛地应用于抗体研究^[5-7]^。本研究利用多肽芯片检测非小细胞肺癌者血清中表皮生长因子受体（epidermal growth factor receptor, EGFR）的自身抗体，并确定其识别的抗原表位，为进一步研究EGFR自身抗体与肺癌的早期诊断和预后之间的关系提供线索。

## 1 材料与方法

### 1.1 材料

#### 1.1.1 血清

肺癌患者血清共20例，其中腺癌和鳞癌各10例，所有肺癌患者均经术后病理明确诊断，同时取10例健康人血清作为正常对照，所有上述血清均取自北京胸科医院细胞生物学实验室标本库2008年2月-2009年2月收集的标本。

#### 1.1.2 试剂

Fmoc-基团保护的20种天然氨基酸干粉（德国，Intavis公司），1-甲基-2-吡咯烷酮（1-methyl-2-pyrrolidone，NMP，Sigma公司），溴酚蓝（Bromphenol blue，BPB，Sigma公司），1-羟基苯并三唑（Hydroxybenzotriazole，HOBt，上海吉尔生化），二异丙基碳二亚胺（Diisopropyl carbodiimide，DIC，Sigma公司），二甲基甲酰胺（Dimethylformamide，DMF，Sigma公司），乙酸酐（Acetic anhydride，国药集团），哌啶（Piperidine，国药集团），乙醇（Ethanol，Sigma公司），三氟乙酸（Trifluoroacetic acid，TFA，Sigma公司），三异丙基硅烷（Triisopropylsilane，Sigma公司），二氯甲烷（Dichlormethane，DCM，Sigma公司）。

#### 1.1.3 仪器

多肽芯片合成仪ASP SL（德国，Intavis公司）

### 1.2 方法

#### 1.2.1 EGFR蛋白胞外段肽库合成

此肽库共包括204个肽段，每个肽段的长度为12个氨基酸（aa），相邻两个肽段之间重叠9个氨基酸，使用Fmoc-保护的20种氨基酸，按照Intavis公司ASP SL多肽芯片合成仪说明书进行多肽合成，将该204个肽段按6行×34列的顺序合成到纤维素膜上，即构成了多肽芯片。

#### 1.2.2 多肽芯片质量控制

多肽芯片合成过程中，每完成一个循环均用乙酸酐进行Caping，封闭未反应的NH2-基团；同时用哌啶Deprotecting脱去Fmoc-保护基团，使被保护的NH2-基团暴露，以便进行下一个循环；哌啶Deprotecting之后，用染色剂溴酚蓝（BPB）进行染色，以确定未反应的NH2-基团已被封闭同时被保护的NH2-基团已经游离，以便进行下一个循环。如此完成12个循环。待芯片合成结束后进行紫外照相，进一步观察芯片合成质量。

#### 1.2.3 混合血清中EGFR自身抗体的检测

使用EGFR多肽芯片分别检测8例肺癌患者混合血清和10例健康对照者混合血清，以确定EGFR自身抗体在肺癌患者血清中是否存在。步骤如下：首先将膜水化（100% ETOH×10 min→75% ETOH×10 min→50% ETOH×10 min→PBS×30 min），用含有0.2% Tween-20和5%脱脂牛奶的PBST溶液室温封闭3 h，按照1:1 000的比例稀释混合血清与多肽芯片共同孵育，4 ℃孵育过夜，PBST洗膜20 min×4次，抗人HRP-标记山羊抗人IgG（1:10 000稀释）室温孵育2 h，最后用PBST洗膜15 min×3次，PBS洗膜15 min×3次，ECL（普利莱公司）化学发光检测。

#### 1.2.4 检测EGFR自身抗体识别的抗原表位

使用EGFR多肽芯片分别检测20例肺癌患者血清，以确定EGFR自身抗体在多肽芯片上所识别的特异性位点即抗原表位。步骤同混合血清检测。

### 1.3 数据分析

用Excel进行数据整理。高频位点的确定，以频率（F） > 2×中位数（Md）为判断依据。

## 2 结果

### 2.1 质量控制

在合成过程中，每一个循环去保护后经溴酚蓝染色发现：芯片背景无着色；芯片上的点着色均匀﹑无重叠，质量良好。芯片合成后紫外照相，结果如图 1所示，共204各点，以6行×34列的方式排列，单点圆形无缺失，点和点之间无重叠，符合实验要求。

**1:**
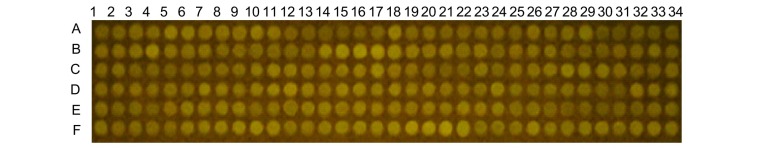
EGFR多肽芯片合成后紫外照相结果。 UV photogram after peptide-array of EGFR synthesized. The library consists of 12 amino acids long peptides with 9 amino acids overlapping with the adjacent peptides immmobilised on a cellulose membrane. Letters on left-side hand of the array indicate rows. Numbers on the upper side of the array indicate columns.

### 2.2 混合血清检测结果

芯片检测结果显示在肺癌患者混合血清中存在EGFR自身抗体，而在健康对照者混合血清中不存在EGFR自生抗体。如图 2A所示黑色位点即为肺癌患者混合血清中EGFR自生抗体所识别的抗原表位即阳性位点；而在健康对照者混合血清中则无阳性位点存在如图 2B所示。

**2:**
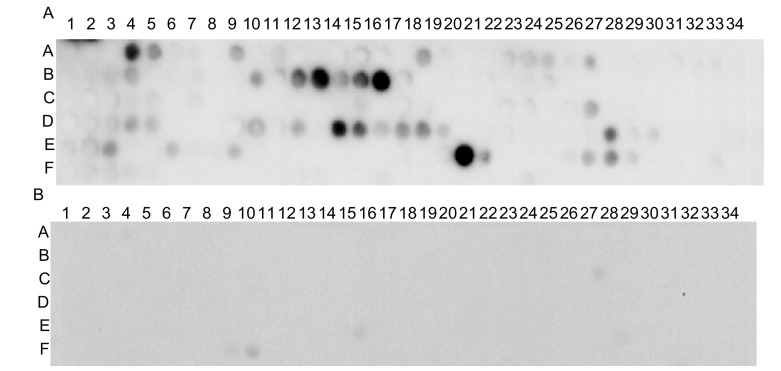
混合血清检测结果。A：8例肺癌混合血清检测结果，图中黑色位点即为阳性位点；B：10例健康人混合血清检测结果，无阳性位点。 The result of mixed serums which is tested by peptide-array. A: The result of mixed serums of 8 NSCLC patients which is tested by peptide-array; B: The result of mixed serums of 10 nomral subjects which is tested by peptide array.

### 2.3 EGFR自身抗体识别的抗原表位

使用EGFR多肽芯片检测20例肺癌患者血清，结果发现有6例阳性，阳性率30%，其中4例腺癌，2例鳞癌。将该6例阳性患者血清中EGFR自身抗体所识别的抗原表位作成频率分布图，如图 3所示，结果发现有9个高频位点存在，该9个高频位点的频率（F） > 2×中位数（Md=16.66%）。其中A9、D4、D10、D13、E29的频率为33.33%；D14、E28的频率为50%；E9、E27的频率为66.67%。9个高频位点在EGFR蛋白胞外段的位置分布如图 4所示，高频位点主要集中在EGFR蛋白胞外段的第Ⅲ和第Ⅳ结构域，此外第Ⅰ结构域也有1个高频位点出现。位于第Ⅲ结构域的高频位点为D4、D10、D13、D14、E9；位于第Ⅳ结构域的高频位点为E27、E28、E29；位于第Ⅰ结构域的高频位点为A9。高频位点所对应的多肽序列即相应的抗原表位见表 1。

**3:**
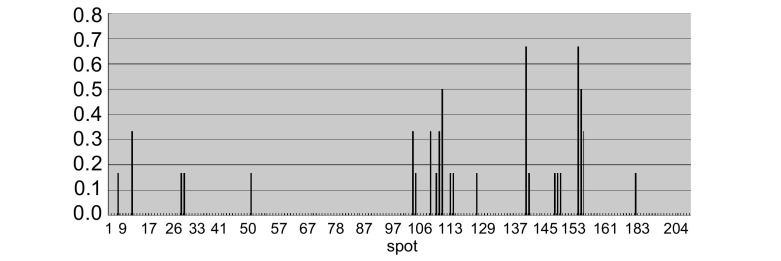
阳性位点频率分布图。将多肽芯片上的204个点作为横坐标，将这些位点在6例阳性患者中出现的频率作为纵坐标。若一位点若在6例阳性患者中均出现则频率为100%，一位点若在6例阳性患者中均不出现则频率为0.00%。 Frequency distribution of positive spots. Abscissa is the 204 sites in peptide array, Ordinate is the frequencies of the 204 sites in the 6 positive patients. One site is positive in all 6 patients, its frequency is 100%; One site is negative in all 6 patients, its frequency is 0.00%.

**4:**
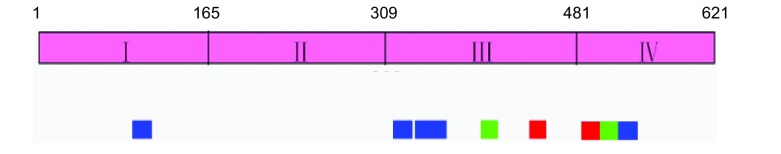
高频位点在EGFR胞外区位置分布图。EGFR胞外区共包括621个氨基酸，分成Ⅰ﹑Ⅱ﹑Ⅲ﹑Ⅳ 4个结构域
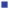
代表的频率为33.33%, 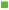
代表的频率为50%, 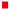
代表的频率为66.67%。 Distribution of high frequency spots in the extracellular domain of EGFR. The extracellular domain of EGFR consists of 621 amino acids and includes Ⅰ, Ⅱ, Ⅲ, Ⅳ structural domains. The frequency of 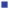 is 33.33%, The frequency of 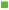 is 50%, The frequency of 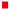 is 66.67%.

**1:** EGFR自身抗体识别的高频位点及其对应的多肽序列 The high frequency spots and the sequence of these spots Spot Sequence of peptide Frequency

Spot	Sequence of peptide	33.33%
A9	Frequency	33.33%
D4	IGIGEFKDSLSI	33.33%
D10	HFKNCTSLSGDL	33.33%
D13	GDLHILPVAFGR	33.33%
D14	HILPVAFRGDSF	50.00%
E9	SDGDVIISGNKN	66.67%
E27	SPEGCWGPEPRD	66.67%
E28	GCWGPEPRDCVS	50.00%
E29	GPEPRDCVSCRN	33.33%
The column of “spot” in the table indicates the number of the spots in peptide-array; The column of “sequence of peptide” in the table indicates the composition of amino acids of the spots with high frequency; The column of “frequency” in the table indicates the frequency of the spots with high frequency in positive patients.		

## 3 讨论

非小细胞肺癌患者约占肺癌患者总数的80%，绝大部分患者在就诊时已处于晚期，因而发展新的非小细胞肺癌早期诊断方法便成为解决这一问题的关键。研究^[8]^显示临床上无法发现的早期肿瘤即可以出现血清中自身抗体阳性，因而血清自身抗体有望作为肺癌早期诊断的分子标志。

EGFR在肿瘤的发生、发展和侵袭中发挥了重要作用，研究^[9]^表明多种人类肿瘤的发生与人体EGFR的过度表达有关，Nakamura等^[10]^进行了大量的回顾性分析发现在39%的肺腺癌和58%的肺鳞癌中存在EGFR的过度表达，过度表达的EGFR为肿瘤的生长提供了生存信号使肿瘤避免凋亡。1996年，Glushkov等^[11]^发现在肺癌病人血清中存在EGFR自身抗体；随后Bei等^[12]^的研究显示不仅在肺癌病人血清中存在EGFR自身抗体，在乳腺癌、胃癌、前列腺癌病人血清中也存在着EGFR自身抗体，这些研究表明EGFR自身抗体存在于多种肿瘤中。然而上述研究只是简单地检测了EGFR自身抗体的有无，并未对EGFR自身抗体所识别的抗原表位进行研究，因而其对基础科研和临床应用的意义有限。

本研究使用EGFR多肽芯片对非小细胞肺癌患者血清中的EGFR自身抗体进行研究，不仅能检测EGFR自身抗体是否存在，同时还能确定EGFR自身抗体所识别的抗原表位，为进一步研究EGFR自身抗体的功能提供了新的依据。混合血清检测结果显示在肺癌患者血清中存在EGFR自身抗体，而在健康对照者血清中不存在EGFR自身抗体，这与Bei等^[8]^使用Western方法得到的结果相同，这说明多肽芯片这一方法可以用于非小细胞肺癌患者血清中EGFR自身抗体的检测，同时也提示EGFR自身抗体有望作为非小细胞肺癌的标志物用于肺癌的早期诊断。

EGFR多肽芯片无论是在检测肺癌患者混合血清还是检测肺癌患者单个血清时，均发现了EGFR自身抗体所识别的抗原表位，目前关于EGFR自身抗体研究的报道很少，而关于EGFR自身抗体所识别抗原表位的研究这应该还是第一次，因而EGFR自身抗体所识别的抗原表位的发现将对后续的研究提供新的线索。

单个血清检测结果发现在20例非小细胞肺癌患者中有6例阳性，EGFR自身抗体在这6例阳性患者中所识别的抗原表位不完全相同，但是也有一些抗原表位被这些阳性患者同时识别，因而便产生了一些高频位点。这9个高频位点在6例阳性肺癌患者中出现的频率为33.33%-66.67%，高频位点的存在提示在EGFR蛋白胞外区存在着一些重要的功能位点，阻断这些功能位点就有可能影响EGFR信号转导通路的功能。通过做高频位点结构分布图发现这些高频位点主要集中在EGFR蛋白胞外段的第Ⅲ和第Ⅳ结构域，此外第Ⅰ结构域也存在1个高频位点。晶体结构研究^[13, 14]^发现EGFR与其配体的结合部位主要由EGFR胞外区的第Ⅰ和Ⅲ结构域组成；第Ⅳ结构域主要调节第Ⅱ结构域的功能，而第Ⅱ结构域为EGFR胞外段的二聚化臂。高频位点集中在这些结构域，可能是通过影响这些区域的功能，而进一步影响EGFR信号转导通路的功能。高频位点和高频位点集中分布这两个现象的发现，为我们进一步研究EGFR和EGFR自身抗体的功能提供了新的线索。

综上所述，本研究利用多肽芯片检测到了肺癌患者血清中存在EGFR自身抗体，同时发现了EGFR自身抗体识别的高频位点和高频位点的集中分布，为进一步研究EGFR自身抗体的作用机制提供了理论基础，同时也为疫苗的研发指明了方向，随着研究的不断深入EGFR自身抗体将对肺癌的诊断﹑治疗和预后评价提供更大的帮助。
